# Emerging Potential of the Phosphodiesterase (PDE) Inhibitor Ibudilast for Neurodegenerative Diseases: An Update on Preclinical and Clinical Evidence

**DOI:** 10.3390/molecules27238448

**Published:** 2022-12-02

**Authors:** Efthalia Angelopoulou, Efstratios-Stylianos Pyrgelis, Christina Piperi

**Affiliations:** 1 1st Department of Neurology, School of Medicine, National and Kapodistrian University of Athens, Eginition Hospital, 11528 Athens, Greece; 2 Department of Biological Chemistry, Medical School, National and Kapodistrian University of Athens, 11527 Athens, Greece

**Keywords:** ibudilast, PDE inhibitor, neurodegeneration, microglia, autophagy, lysosomes

## Abstract

Neurodegenerative diseases constitute a broad range of central nervous system disorders, characterized by neuronal degeneration. Alzheimer’s disease, Parkinson’s disease, amyolotrophic lateral sclerosis (ALS), and progressive forms of multiple sclerosis (MS) are some of the most frequent neurodegenerative diseases. Despite their diversity, these diseases share some common pathophysiological mechanisms: the abnormal aggregation of disease-related misfolded proteins, autophagosome–lysosome pathway dysregulation, impaired ubiquitin–proteasome system, oxidative damage, mitochondrial dysfunction and excessive neuroinflammation. There is still no effective drug that could halt the progression of neurodegenerative diseases, and the current treatments are mainly symptomatic. In this regard, the development of novel multi-target pharmaceutical approaches presents an attractive therapeutic strategy. Ibudilast, an anti-inflammatory drug firstly developed as an asthma treatment, is a cyclic nucleotide phosphodiesterases (PDEs) inhibitor, which mainly acts by increasing the amount of cyclic adenosine monophosphate (cAMP) and cyclic guanosine monophosphate (cGMP), while downregulating the pro-inflammatory factors, such as tumor necrosis factor-α (TNF-α), macrophage migration inhibitory factor (MIF) and Toll-like receptor 4 (TLR-4). The preclinical evidence shows that ibudilast may act neuroprotectively in neurodegenerative diseases, by suppressing neuroinflammation, inhibiting apoptosis, regulating the mitochondrial function and by affecting the ubiquitin–proteasome and autophagosome–lysosome pathways, as well as by attenuating oxidative stress. The clinical trials in ALS and progressive MS also show some promising results. Herein, we aim to provide an update on the emerging preclinical and clinical evidence on the therapeutic potential of ibudilast in these disorders, discuss the potential challenges and suggest the future directions.

## 1. Introduction

Neurodegenerative diseases constitute a broad range of disorders of the central nervous system (CNS), which are characterized by progressive neuronal degeneration [1]. Alzheimer’s disease (AD), Lewy body disease (LBD), Parkinson’s disease (PD), amyolotrophic lateral sclerosis (ALS), fronto-temporal lobar degeneration (FTLD) and Huntington’s disease (HD) are some of the most frequent neurodegenerative disorders. Multiple sclerosis (MS)—and especially its progressive forms—are also considered to involve a neurodegenerative process [2]. Worldwide, millions of individuals are affected by neurodegenerative disorders, which lead to an important economic and social burden for the patients and caregivers. With the exception of some rare genetic forms, most cases of neurodegenerative disorders are multifactorial, with both the genetic and environmental factors contributing to their development [3,4,5,6].

Each neurodegenerative disease is neuropathologically characterized by the accumulation of specific abnormal proteins in the vulnerable cells and brain regions, accompanied by usually relatively specific clinical symptomatology [1]. Despite their diversity, neurodegenerative diseases share some common pathophysiological underlying mechanisms, such as the abnormal aggregation of disease-related misfolded proteins, autophagosome––lysosome pathway dysregulation, impaired ubiquitin–proteasome system, oxidative damage, mitochondrial dysfunction and excessive neuroinflammation [4,7,8,9,10].

Even though the inflammatory response is a physiological procedure during CNS injury and infections, aberrant neuroinflammation is a shared pathophysiological hallmark of the whole spectrum of neurodegenerative diseases [7,8,9,10,11,12]. The accumulation of abnormal protein aggregates induces microglial activation, which results in the production of pro-inflammatory cytokines, including tumor necrosis factor α (TNF-α), IL-6 and IL-1β [12], mitochondrial impairment [13], lysosomal dysfunction and oxidative stress [14]. Toll-like receptor 4 (TLR4)-mediated pathways and other cytokines including macrophage migration inhibitory factor (MIF) also play major roles in the neuroinflammatory responses in neurodegeneration [8,15].

Emerging evidence demonstrates that cyclic nucleotide phosphodiesterases (PDEs), which degrade cyclic nucleotides—cyclic adenosine monophosphate (cAMP) and cyclic guanosine monophosphate (cGMP)—are majorly implicated in the pathophysiological mechanisms of neurodegenerative diseases [16]. PDEs regulate a wide variety of cellular functions by affecting cyclic nucleotide-mediated signaling, including inflammatory responses, cell survival and apoptosis, neuronal plasticity, the regulation of neurotransmitters and neurotrophic factors, synaptic function, intracellular calcium levels, astrocytic function, oxidative stress, autophagy, and mitochondrial homeostasis [16,17].

There is still no approved disease-modifying treatment for neurodegenerative diseases that could halt the disease’s progression, and the current therapeutic approaches provide only partial and temporary symptomatic relief. Hence, there is a crucial demand for potent treatment approaches that could slow the disease’s progression, maintain functional ability, and prolong the survival of patients with neurodegenerative disorders. Given the several molecular mechanisms and cellular processes implicated in their pathogenesis, the development of a multi-target drug might represent an attractive pharmaceutical candidate.

Ibudilast (3-isobutyryl-2-isopropylpyrazolo-[1, 5-a] pyridine) was firstly developed and currently used in Asian countries as an asthma treatment [18,19]. Ibudilast is a small molecule that can be orally delivered, mainly acting in an anti-inflammatory manner. It is a relatively non-selective inhibitor of several PDEs, including PDE3, PDE4, PDE10 and PDE11, as well as MIF and TLR-4 [20,21,22]. One principal mechanism of ibudilast is the ability to increase the intracellular cAMP levels, thereby affecting multiple signaling pathways and cellular functions [20,21,22].

**Figure 1:**
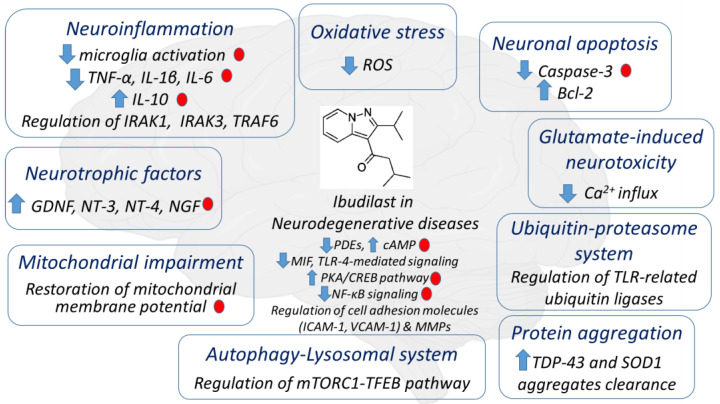
Effects of ibudilast possibly related to neurodegenerative diseases. Ibudilast can suppress neuroinflammation by inhibiting microglia activation, downregulating the pro-inflammatory cytokines TNF-α, IL-1β and IL-6, upregulating the anti-inflammatory cytokine IL-10 and regulating IRAK1, IRAK3 and TRAF6. Ibudilast can upregulate the neurotrophic factors GDNF, NT-3, NT-4 and NGF and prevent mitochondrial impairment by restoring the mitochondrial membrane potential. It can also affect the autophagy-lysosomal system by regulating mTORC1-TFEB pathway. Ibudilast can also promote TDP-43 and SOD1 aggregates clearance, thereby suppressing abnormal protein aggregation. Ibudilast also affects ubiquitin–proteasome system by regulating TLR-related ubiquitin ligase, and it protects against glutamate-induced neurotoxicity by reducing Ca^2+^ influx. Ibudilast also protects against neuronal apoptosis by downregulating caspase-3 and upregulating bcl-2. Ibudilast may also suppress oxidative stress by reducing the production of ROS. Apart from PDEs inhibition, ibudilast can also inhibit MIF and TLR-4. MIF inhibition and subsequent MIF reduction results in the downregulation of its receptor CD74 and AKT expression. TLR-4 blocking may lead to the reduced production of pro-inflammatory cytokines via pathways that also implicate NF-κΒ, IRAK1 and TRAF6. Although it is not fully elucidated which of the above molecular and cellular effects of ibudilast are mediated through PDE inhibition; the small red circles indicate the specific mechanisms that there is some evidence suggesting to be at least partially induced by the inhibition of PDEs.

Apart from its bronchodilator properties, several pharmacological activities have been attributed to ibudilast, including the vasodilating, anti-thrombotic and anti-leukotriene properties [23]. The emerging preclinical evidence has demonstrated that ibudilast may also exert significant anti-neuroinflammatory effects for a wide range of neurological disorders, including chronic cerebral hypoperfusion [24], peripheral and central neuropathic pain [25], opioid withdrawal [26], human immunodeficiency virus-1 (HIV-1)-associated neurocognitive disorders (HAND) [27], cerebral aneurysms [28], transient cerebral ischemia [29], ischemic brain injury [30], post-stroke dizziness [18,19], oxaliplatin-induced tactile allodynia and cognitive impairment [31], tacrolimus-induced neurotoxicity [32] and cocaine use disorder [33], among others. Neuroinflammation and microglia activation are shared mechanisms underlying the pathophysiology of these conditions [34], thus also highlighting the potential of ibudilast to mitigate aberrant inflammatory responses in the case of neurodegenerative diseases. The proposed molecular mechanisms underlying the role of ibudilast in neurodegenerative diseases are depicted in Figure 1.

Based on the above evidence, during the last decade ibudilast has gained increasing attention against neurodegeneration. It has been already investigated in several clinical trials for neurodegenerative diseases including ALS and progressive forms of MS with variable outcomes [35,36], while other clinical trials are still ongoing.

Although the role of ibudilast in neurological disorders has been previously discussed [37], there is no recent review focusing on neurodegenerative diseases. Herein, we aim to provide an update on the emerging preclinical and clinical evidence on the therapeutic potential of ibudilast in these disorders, discuss the potential challenges and suggest the future directions.

## 2. Pharmacology and Mechanism of Action of PDEs and Ibudilast in the CNS

In mammals, the PDEs superfamily is classified into eleven families, known as PDE1—PDE11. These different families vary in their kinetic characteristics, distribution in various tissues, response to molecular regulators and co-factors (Ca^2+^, cGMP, Zn^2+^ and Mg^2+^), specificity for synthetic PDE inhibitors and occasionally their specificity for substrates (cAMP or cGMP) [38,39]. Each PDE family is encoded by one or multiple genes, and alternative mRNA splicing results in several splice variants [39]. To date, more than fifty PDEs have been identified in humans. A numeral following PDE indicates the specific family (e.g., PDE4), the capital letter that follows indicates the gene (e.g., PDE4D), while a numeral at the end shows the splice variant [38,40]. PDEs are widely expressed in human tissues including the CNS [41].

PDEs are majorly implicated in multiple signaling pathways, by reducing the intracellular levels of cAMP and cGMP [42]. In particular, PDEs can catalyze the hydrolysis of the phosphodiester bond of cAMP and cGMP, leading to the production of the inactive AMP and GMP [38]. PDE inhibition can result in increased levels of cyclic nucleotides, which regulate several cellular functions, acting as secondary messengers [38]. Cyclic nucleotides are majorly implicated in the transcriptional regulation via the cAMP response element-binding protein (CREB) [43]. Low amounts of cyclic nucleotides may result in the downregulation of neurotrophic factors, including brain-derived neurotrophic factor (BDNF), nerve growth factor (NGF), neurotrophin-3 (NT-3) and neurotrophin-4 (NT-4) [43]. Higher levels of cAMP may lead to the downregulation of nuclear factor kappa B (NF-κB), thereby resulting in the reduced production of pro-inflammatory cytokines and inducible nitric oxide synthase (iNOS) [44].

Ibudilast acts as a relatively non-selective PDE inhibitor, but its specificity depends on the species, tissue, and cell type. More specifically, ibudilast has been shown to predominantly inhibit PDE3 in the hearts, the kidneys, and the brains of rats, with a lower activity against PDE1, PDE2 and PDE4 [22]. Ibudilast could effectively inhibit PDE2, PDE4 and PDE5 but not PDE3 in human platelets [45], whereas the preferential inhibition of PDE4 [46] or PDE3, PDE4, PDE10 and PDE11 by ibudilast [20] has also been described.

Apart from the PDEs inhibition, ibudilast can also inhibit MIF and TLR-4. MIF inhibition and subsequent MIF reduction may result in the downregulation of its receptor CD74 and AKT expression [20,21,22]. TLR-4 blocking may lead to the reduced production of pro-inflammatory cytokines via pathways that also implicate NF-κΒ, IRAK1 and TRAF6 [8,20,21,22].

Although ibudilast has been used for a long time in Asia, evidence on its pharmacokinetics and pharmacodynamics are rather limited [37]. Ibudilast was shown to be excreted in urine, and to a lesser extent in feces in an in vivo study involving dogs, rats, and monkeys [37]. The incidence of plasma protein binding after oral administration was about 98% in this study [37]. It can be metabolized by several cytochrome P450 isozymes, and its primary metabolite is a 6,7-dihydrodiol metabolite [37]. Ibudilast pharmacokinetics have been investigated in humans after the delivery of 30 mg of it and after multiple doses of 30 mg of it twice daily in healthy subjects: the peak concentrations after a single dose were 32 ng/mL in the plasma, while the elimination half-life was 19 h, and the pharmacokinetics were linear at the dose range of 30–100 mg [47]. Ibudilast at the dose range of 20–50 mg b.i.d. was well tolerated in healthy individuals, although the patients with diabetes exhibited slightly less tolerance to it [47].

Regarding its bioavailability in the CNS, the preclinical evidence has shown that after an oral delivery of ibudilast at 50 mg/kg/day for seven consecutive days, it can be detected postmortem in the central nervous system of rats at elevated concentrations compared to its plasma levels [48], suggesting that it can cross the blood–brain barrier. However, this study demonstrated that its oral bioavailability displayed significant inter-species variability, which was related to the varied expression of first-pass metabolic enzymes and gut transporters across the animal species [48]. Nevertheless, pharmacokinetic and pharmacodynamic in vivo or postmortem human studies investigating the concentration of ibudilast in the cerebrospinal fluid (CSF) or brain tissue are lacking [49].

The preclinical evidence has shown that ibudilast displays anti-inflammatory and neuroprotective properties in the neuronal and glial cells and the animal models of several neurological disorders. The in vitro evidence has shown that it can suppress the activation of microglia and the subsequent release of pro-inflammatory cytokines, including TNF-α [22,44], interleukin (IL)-1β and IL-6 [44], in addition, it can enhance the formation of anti-inflammatory cytokines, such as IL-10 [44]. Ibudilast can also exhibit antioxidant properties since it can prevent the generation of nitric oxide (NO) and reactive oxygen species (ROS) in the microglia in vitro [44]. In addition, it may display a neuroprotective role in vitro by preventing microglial activation-induced neuronal cell death and promoting the production of neurotrophic factors, including neurotrophin (NT)-4, nerve growth factor (NGF) and glia-derived neurotrophic factor (GDNF) in the activated microglia [44]. The microglial generation of chemokine monocyte chemoattractant protein-1 (MCP-1) was also reduced by the ibudilast treatment [37]. Apart from the neurons and microglia, ibudilast also affects the oligodendrocytes and astrocytes. In particular, the in vitro evidence shows that it can attenuate oligodendrocyte cell toxicity induced by kainite and astrocyte apoptotic cell death induced by reperfusion [37].

Hence, the accumulating evidence suggests that ibudilast may act in an anti-inflammatory, antioxidant, and potentially neuroprotective manner, at least by suppressing microglial activation, the upregulating neurotrophic factors, preventing neuronal and astrocytic cell death and inhibiting oxidative stress. Hence, given its multi-target mechanism of action, it represents a promising pharmaceutical candidate against neurodegenerative diseases.

In the following sections, we will discuss the preclinical and clinical evidence on the implication of ibudilast in the most common neurodegenerative diseases, the therapeutic challenges, and the potential future directions.

### 2.1. Alzheimer’s Disease

AD is the most prevalent form of dementia. Its main clinical feature is gradual cognitive decline, including impaired memory, visuospatial, language and executive function, while at the later stages, the patients display behavioral symptoms resulting in significant functional disability [50]. The neuropathological AD hallmarks of AD are the deposition of the extracellular plaques of amyloid-beta and the intracellular neurofibrillary tangles of the abnormally phosphorylated tau protein [1]. Although the exact pathogenic molecular mechanisms of AD have not been identified, amyloid-beta pathology is closely related to the dysregulation of apoptosis, calcium homeostasis, oxidative stress, glutamate-induced neurotoxicity and aberrant neuroinflammatory responses [51,52].

cAMP and cGMP play key roles as secondary messengers in the human brain, affecting long-term memory and synaptic plasticity [53]. These two molecules can activate CREB, thereby enhancing gene transcription. cAMP and cGMP are also implicated in the production of amyloid-beta [54], and the upregulation of cAMP signaling can suppress neuroinflammatory responses and apoptosis by suppressing the activation of caspase-3 [34,55]. The PDE8B levels were elevated in the cortex and hippocampus of AD patients at Braak stages III-VI [42], and PDE3 was upregulated in the cerebral blood vessels of postmortem human brain tissue of patients with AD [56]. This evidence suggests that PDE inhibitors might exert a protective role against AD pathology.

Indeed, the preclinical evidence has demonstrated that PDE inhibitors may exert beneficial effects in AD. In this regard, the PDE4 inhibitor rolipram could reverse Aβ-induced cognitive impairment at least by regulating the neuroinflammatory and apoptotic responses in rats mediated by cAMP/CREB signaling [55]. Zatomilast (BPN14770), a PDE4D inhibitor, has been shown to improve memory, prevent the loss of dendrites and spine density as well as inhibit the amyloid-beta-induced reduction of CREB, BDNF and NGF in the hippocampus of mice models of AD [57]. Furthermore, cilostazol, a PDE3 inhibitor, could prevent amyloid-beta-induced oxidative stress and memory impairment [58], as well as APOE-mediated amyloid-beta aggregation in mice [59]. Cilostazol was also able to promote proteasome-mediated proteolysis, suppress tauopathy and attenuate cognitive impairment in vivo [60]. The in vitro evidence has also demonstrated that cilostazol could regulate autophagy by upregulating SIRT1, and subsequently, enhance amyloid-beta clearance and cell viability [61].

Based on this promising in vitro and in vivo evidence, MK0952, a PDE4 inhibitor, had been tested for patients with mild-to-moderate AD in a phase 2 randomized clinical trial (NCT00362024). In AD, zatomilast has been investigated in phase 1 clinical trials (NCT02648672, NCT02840279, NCT03030105) as well as in a phase 2 clinical trial (NCT03817684) [38,62]. A randomized, placebo-controlled phase 4 clinical trial has been completed, which aimed to evaluate the efficacy of cilostazol in AD patients (mild-to-moderate stages) with subcortical white matter hyperintensities (WMHI) treated with donepezil (NCT01409564). This study demonstrated that cilostazol added to a donepezil treatment could slow the regional cerebral metabolism decline in AD with white matter lesions compared to donepezil alone which could not [63].

The in vitro evidence has demonstrated that ibudilast could protect against glutamate-induced neurotoxicity and increase the intracellular cAMP levels in cultured hippocampal neurons from rats [23]. The ibudilast treatment was also associated with the decreased glutamate induced Ca^2+^ influx [23]. This evidence suggests that the inhibition of glutamatergic neurotoxicity is another potential mechanism underlying the beneficial effects of ibudilast in neurodegenerative diseases, including AD.

The in vivo evidence has demonstrated that ibudilast was able to effectively reverse the lipopolysaccharide (LPS)- and interferon-gamma (INF-γ)-induced inhibition of long-term potentiation (LTP) in the CA1 hippocampal region of rat models [44]. Although the underlying molecular mechanism was not investigated in this study, it was proposed that the protein kinase A (PKA)/CREB signaling pathway might play a significant role [44]. This hypothesis was based on the fact that rolipram, a type IV-specific PDE inhibitor, could reverse the amyloid beta-induced inhibition of LTP and the downregulation of the PKA/CREB pathway in hippocampal neuronal cultures in another study [64].

Moreover, an in vivo study showed that the ibudilast pretreatment was associated with the prevention of amyloid-beta-induced memory and spatial learning impairment as well as neurotoxicity in mouse models of AD [34]. In particular, ibudilast could act in an anti-inflammatory and anti-apoptotic manner by suppressing the generation of pro-inflammatory cytokines, including NF-κB p65 and TNF-α, preventing the activation of the pro-apoptotic caspase-3, as well as hindering the downregulation of the anti-apoptotic protein Bcl-2 in the cortex and hippocampus of amyloid-beta-injected mice [34].

Interestingly, a recent study was conducted using a multi-scale predictive modeling framework, which integrated machine learning, systems pharmacology, and biophysics in order to screen potential drugs for AD based on tissue samples [65]. This study showed that ibudilast had significant repurposing potential for AD [65]. An in vivo study by the same authors indicated that a long-term ibudilast treatment was associated with reduced hippocampal-dependent spatial memory impairment, hippocampal amyloid-beta plaque deposition and tau paired-helical filament burden as well as microgliosis in Fisher transgenic 344-AD rats [65]. The RNA sequencing of the hippocampal samples showed that ibudilast could affect the expression of the TLR as well as the ubiquitin–proteasome pathways in the rat models of AD in this study [65]. Furthermore, ibudilast could downregulate the activity of interleukin 1 receptor associated kinase 1 (IRAK1) by elevating the expression of interleukin-1 receptor-associated kinase 3 (IRAK3), and also affecting the levels of tumor necrosis factor receptor (TNFR)-associated factor 6 (TRAF6) and possibly other TLR-related ubiquitin ligases [65]. Therefore, ibudilast may serve as a promising candidate targeting multiple signaling pathways in AD including TLR-mediated signaling and the ubiquitin–proteasome system.

To date, there has been no clinical trial of ibudilast in AD. However, given the emerging preclinical evidence on the impact of ibudilast in AD-related pathways and pathophysiology, combined with its beneficial effects at least in animal models, it represents a novel drug whose role in AD in humans needs to be further investigated.

### 2.2. Parkinson’s Disease

After AD, PD is the second most frequent neurodegenerative disorder. Its main characteristic is the loss of the dopaminergic neurons in the substantia nigra pars compacta (SNpc), which is accompanied by the subsequent nigrostriatal degeneration. PD patients experience both motor and non-motor clinical manifestations, such as bradykinesia, resting tremor, postural instability, depressive and other psychiatric symptoms, autonomic dysfunction and cognitive decline. The neuropathological hallmark of PD is the abnormal deposition of Lewy bodies that contain alpha-synuclein. Most PD cases are sporadic, while some cases are caused by gene mutations in the gene encoding α-synuclein (SNCA), Parkin, PINK1 and others. The dysregulation of the autophagy and lysosomal pathways, apoptosis, mitochondrial dysfunction and excessive neuroinflammation are some of the major pathophysiological underlying mechanisms. Dopaminergic agents are the gold standard for the treatment of PD, although they provide temporary symptomatic management; in this regard, neuroprotective and disease-modifying agents are needed. Nigrostriatal dysfunction has been associated with downregulation of the cAMP-mediated signaling pathway [43]. PDE1, PDE2, PDE4 and PDE10 are abundantly expressed in the striatum [66] (Table 1). In particular, PDE10A is found at high levels in the GABAergic medium spiny neurons, and it may play an important role in PD [67]. Rolipram, a PDE4 inhibitor, displayed neuroprotective properties in the 1-methyl-4-phenyl-1,2,3,6-tetrahydropyridine (MPTP) mouse models of PD since it could inhibit MPTP-induced dopamine loss in the striatum and prevent dopaminergic neuronal loss in the SN of the animals [68]. Furthermore, PDE4 inhibition by FCPR16 could prevent the MPP+-induced reduction of oxidative stress and the potential of the mitochondrial membrane [69]. In addition, PDE4 inhibition could stimulate AMPK-dependent autophagy in SH-SY5Y cells in vitro [17]. Hence, PDE inhibitors may act in a neuroprotective manner in PD by inducing autophagy and protecting against mitochondrial impairment and oxidative damage.

An in vivo study has shown that pretreatment with ibudilast was associated with reduced astroglia activity and increased GDNF in the striatum of MPTP mouse models of PD. Ibudilast could also suppress the production of pro-inflammatory cytokines, including IL-6, IL-1β and TNF-α [66]. However, ibudilast did not alter the dopaminergic neuronal cell survival and tyrosine hydroxylase levels in the striatum seven days after the acute MPTP insult in this study [66]. Based on these results, it is suggested that the beneficial effects of ibudilast could be due to its action in non-dopaminergic cells, such as the medium spiny neurons [66], although further evidence is needed to confirm this hypothesis.

Although dopaminergic drugs—and mainly levodopa—are very effective in improving the motor symptoms of PD, long-term levodopa use often results in the development of involuntary movements, known as levodopa-induced dyskinesias. Interestingly, cAMP and cGMP have been shown to be dysregulated during levodopa-induced dyskinesias in the 6-hydroxydopamine-treated hemi-parkinsonian rat models of PD [70]. These effects were partially prevented by the pretreatment with zaprinast, a PDE inhibitor [70]. The role of ibudilast in levodopa-induced dyskinesias remains unknown.

Like AD, there has been no clinical trial testing the safety and effectiveness of ibudilast in PD or levodopa-induced dyskinesias. Given its pleotropic effects, further preclinical and clinical evidence are needed in order to elucidate its specific efficacy in the case of PD too.

### 2.3. Amyotrophic Lateral Sclerosis

ALS is one of the most devastating neurodegenerative disorders, with there being no available therapy that could halt or significantly delay of the disease’s progression. After the onset of the clinical symptoms, the median survival time of patients with is approximately 30 months [35]. In ALS, the upper and lower motor neurons progressively degenerate, leading to voluntary muscle weakness, which is accompanied by dysphagia, dysarthria and breathing difficulties. Currently, riluzole and edaravone remain the only approved drugs for ALS, which show modest effectiveness in slowing the progression of the disease, and they are not also available in all countries [71].

Multiple pathways and mechanisms, including axonal damage, abnormal protein accumulation, oxidative stress, mitochondrial dysfunction, excitotoxicity and neuroinflammation are implicated in the pathogenesis of ALS [72]. TAR DNA binding protein (TDP-43) and mutant superoxide dismutase 1 (SOD1) are key proteins that form aggregates in ALS [73,74]. The dysregulation of the ubiquitin–proteasome and autophagosome–lysosome systems may result in impaired TDP-43 and SOD1 degradation, leading to the formation of TDP-43 and SOD1 aggregates [75,76].

The accumulating preclinical and clinical evidence demonstrate that neuroinflammation is highly implicated in ALS pathophysiology [72,77]. An activated microglia results in excessive inflammatory responses, via TLR-2 and TLR-4, and scavenger receptor-mediated pathways in, in vitro and in vivo models of ALS [77]. The patients with a more rapid progressive course and more severe clinical upper motor neuron deficits show increased microglial activation in their corticospinal tract [78]. The [11C]PBR28 radio ligand can bind to the translocator protein (TSPO), a protein that is found in high amounts in reactive astrocytes and activated microglia [79]. Ιncreased activation of microglia has been detected in the precentral and paracentral gyri of patients with ALS compared to that of healthy controls which was assessed by [11C]PBR28-PET [80]. The increased activation of the microglia in the motor cortex has been associated with worse clinical symptoms which was assessed by the fine motor subscale of revised ALS functional rating scale (ALSFRS-R) and the Upper Motor Neuron Burden (UMNB) [80].

The preclinical evidence has demonstrated that ibudilast may have a therapeutic potential in ALS. A recent in vitro study has shown that an ibudilast treatment could stimulate the clearance of aggregates of TDP-43 and SOD1 in HEK293 and NSC-34 cells, which are used for modelling motor neuron neuronal cells [81]. The underlying mechanism involved the ibudilast-mediated induction of autophagy, an increase in the number of autolysosomes and an enhancement of lysosomal biogenesis through the enhancement of the nuclear translocation of transcription factor EB (TFEB) and the downregulation of the mammalian target of rapamycin complex 1 (mTORC1) [81]. In this study, ibudilast was also able to prevent TDP-43-induced neurotoxicity in NSC-34 cells [81]. Therefore, ibudilast may act neuroprotectively in the cellular models of ALS by enhancing autophagy and lysosomal biogenesis via its implication in the mTORC1-TFEB signaling pathway.

A placebo-controlled randomized Phase 1b/2a clinical trial (NCT02238626) investigating the tolerability, safety, and clinical effectiveness of ibudilast (60 mg/day) as an adjunct therapy to the standard riluzole treatment in 60 ALS patients has been completed [82]. In the early cohort, ibudilast was safe and well-tolerated over a twelve-month period [82]. No significant difference in the clinical progression was detected between the ibudilast and placebo groups, which was assessed by the hand-held dynamometry, ALS Functional Rating Scale-revised (ALSFRS-R) and the ALS Assessment Questionnaire-5 items (ALSAQ-5) [35]. However, a further subgroup analysis demonstrated that ibudilast might provide more benefits to the ALS patients with upper limb or bulbar onset, and it could possibly, delay the progression of the disease if it is administered at an early stage, particularly if the onset of the symptoms at the point of screening is less than 17.1 months [35].

An open-label phase 1b clinical trial (NCT02714036) in 35 ALS patients showed that an ibudilast treatment for 36 weeks at high dose (up to 100 mg daily) failed to reduce neuroinflammation and axonal loss [49]. In particular, ibudilast was ineffective in inhibiting microglia activation in the primary motor cortex of ALS patients, which was evaluated by PBR28-PET in 12–24 weeks, and as well as this, the serum neurofilament light chain (NfL) levels, an indicator of neuronal axonal loss, remained unchanged over 36–40 weeks [49]. Importantly, most of the participants displayed at least one potentially ibudilast-related adverse event: about one-third of the patients required a dosage reduction, while about another one-third discontinued ibudilast treatment because of ibudilast-related adverse events [49]. Although no serious adverse events were associated with the ibudilast treatment, the investigational drug was not well tolerated at this high dose, with the most frequent adverse events being nausea, fatigue, diarrhea, insomnia, and other gastrointestinal symptoms [49].

There is an ongoing phase 2b/3 double-blind randomized placebo-controlled multicenter clinical trial (COMBAT-ALS, NCT04057898) which aims to assess the tolerability, safety, and effectiveness of ibudilast (up to 100 mg/day) for twelve months in ALS patients, which is being followed by an extension open-label phase for six months [35]. The primary outcome measure will be the ALSFRS-R score change from the baseline to month 12, which is widely used for the assessment of the functional status of ALS patients. The secondary outcomes are the change from the baseline of muscle strength as evaluated by hand-held dynamometry, their quality of life and the time to survival, among others.

Collectively, the preclinical evidence shows that ibudilast may exert a protective role in ALS by regulating autophagy and lysosomal function. In addition, the ibudilast treatment is associated with some potential clinical benefits in specific ALS subgroups, such as those at the early stages and the upper limb or bulbar onset stages. Ibudilast at the high dose of 100 mg/day may be not as well tolerated in ALS patients. Nevertheless, the results of the more prolonged clinical trial that is still ongoing will provide further evidence about the effectiveness and tolerability of ibudilast in ALS.

### 2.4. Multiple Sclerosis

MS is a chronic autoimmune and neurodegenerative CNS disorder, representing one of the major causes of disability among young adults. Its core characteristic is the demyelinated lesions in the brain and spinal cord; it is considered to be a multifactorial disorder since genetic and environmental factors contribute to its development [83]. The classic types of the disease are the following: relapsing remitting, primary progressive, secondary progressive and progressive relapsing. The most frequent type is relapsing remitting MS, during which the patients experience periods of clinical attacks, while at the later stages, MS often follows a progressive course [83]. Primary progressive MS is a rarer MS type, in which patients develop a progressive course from the disease’s onset. Although there are several disease-modifying treatments for relapsing-remitting multiple sclerosis (interferon-β 1a and 1b, glatiramer acetate, natalizumab, fingolimod and teriflunomide, among others), the therapeutic choices for the progressive forms of MS are very limited [83].

MS is a primarily inflammatory disorder, and the dysregulation of immune and inflammatory responses is a key underlying mechanism. The inflammatory demyelinating process is a hallmark of the MS early stages, whereas progressive neuronal and axonal degeneration coexist and majorly contribute to the disability and cognitive impairment of the patients at the later stages [84]. The studies using experimental autoimmune encephalomyelitis (EAE), which is broadly used as an animal model for MS in humans, have shown that the dysregulation of T-cells, microglia and B-cells-mediated signaling are implicated in its pathogenesis [84]. Relapsing MS is pathophysiologically characterized by periods of peripheral activation of the immune cells and their infiltration to the CNS, resulting in focal injury of the white matter. However, progressive MS is considered to be caused by low-grade, chronic and rather multifocal neuroinflammation in the CNS regions, including in the leptomeninges and Virchow-Robin spaces, progressively leading to neuronal injury and possibly, neurodegeneration [85].

The clinical evidence has demonstrated that MIF levels are higher in the cerebrospinal fluid (CSF) of the patients with progressive MS compared to those with non-progressive forms; further, primary progressive MS patients exhibit increased levels of serum TNF-α [86]. TLR-2, TLR-4 and their ligand high mobility group box chromosomal protein 1 (HMGB1) are highly expressed in the resident microglia and activated macrophages of active MS lesions and EAE [87,88]. Since the PDE inhibitors and ibudilast in particular may act in an anti-inflammatory manner by inhibiting MIF, TNF-α and TLR-4, they have been suggested to represent an attractive candidate for MS treatment.

The initial evidence about the role of PDE inhibitors in MS comes from an in vivo study in 1995 using rolipram, a PDE4 inhibitor [89]. In this study, rolipram could prevent the clinical signs of demyelination in EAE rat models [89]. Rolipram was also associated with the reduced production of pro-inflammatory cytokines and mainly TNF-α in human myelin basic protein (MBP)-specific T cells [89]. Another in vivo study indicated that TNF-α levels were reduced, as well as the clinical signs of MS, and the neuroimaging abnormalities on an MRI were prevented in marmoset models of EAE treated with rolipram [90] (Table 2). Furthermore, the preventive—but not the therapeutic—administration of rolipram could ameliorate EAE in rats in another study [91]. Given this evidence, rolipram was tested at a clinical level; however, the clinical trial with rolipram in patients with MS was prematurely stopped since the drug was not well tolerated, and the patients demonstrated increased contrast-enhanced brain lesions on the MRIs [92].

Concerning ibudilast, the in vivo evidence has shown that an ibudilast pretreatment could prevent EAE in rats, although it could not alter the clinical course when it was administered after the onset of the disease [93]. Additionally, an ibudilast pretreatment was associated with a lessened neuroinflammatory response in the spinal cord, a mild inhibition of MBP-induced T cell proliferation in the lymph nodes, a reduced release of IFN-γ from the T cells and a decreased secretion of TNF-α from the macrophages [93]. This study demonstrated the therapeutic potential of ibudilast in animal models of MS, thus paving the way for its clinical testing in humans. However, the fact that rolipram and ibudilast were not effective after the onset of the clinical signs of the disease in two of the abovementioned studies [34,91] highlights the importance of the timing of their administration in order for them to be effective. Hence, it can be hypothesized that the use of ibudilast at the earliest stages may be required for its clinical efficacy.

A phase 2 randomized placebo-controlled clinical trial has demonstrated that ibudilast (30 to 60 mg/day) could not inhibit the formation of new active magnetic resonance imaging (MRI) lesions or clinical relapses in relapsing MS patients [94]. However, a post-hoc analysis of this study demonstrated that ibudilast was associated with a slower rate of clinical progression, and a reduced ratio of gadolinium-enhancing lesions being converted into persistent hypointense black holes on the T1-weighted MRI images, which are thought to represent the severe injury of the neuronal tissue [94]. Hence, it has been proposed that ibudilast may not be able to act in an anti-inflammatory fashion in relapsing MS, but it could rather act neuroprotectively by preventing continuous neuronal damage after inflammatory tissue injury [95].

Based on the results of the abovementioned clinical trial, it has been hypothesized that ibudilast might be beneficial in progressive forms of MS, which are characterized by accumulating lesions of potentially irreversible neuronal damage. In this regard, a phase 2 randomized placebo-controlled clinical trial (SPRINT-MS, NCT01982942) has shown that ibudilast (up to 100 mg/day) over a period of 96 weeks was associated with a slower progression of the whole-brain atrophy and gray matter atrophy of the patients with primary or secondary progressive MS [18,36]. However, ibudilast was not associated with fewer enlarging or new T2-weighted or new T1-weighted MRI lesions [36]. The ibudilast treatment could also potentially attenuate retinal thinning which was evaluated by optical coherence tomography (OCT) [96]. However, the disability progression was shown to be similar between the ibudilast and placebo groups in this study over the same period [18]. In addition, no significant alterations in the neurofilament light (NfL) levels in the serum and CSF (as a marker of neuroaxonal injury) were detected between the two groups [95]. The most common adverse events in this study were gastrointestinal complains, headaches and depressive symptoms [18].

A post hoc analysis of this clinical trial demonstrated that the overall treatment effect of ibudilast in brain atrophy was mainly driven by the patients with primary progressive MS and not secondary progressive MS [97]. These observed differences have been attributed to the more rapid progression of brain atrophy in the primary progressive MS placebo subgroup compared to the secondary progressive MS placebo subgroup [97], which has also been previously described [98]. Based on these observations, it can be hypothesized that ibudilast might be more beneficial in the cases with a more rapid or aggressive neurodegenerative process and highlights the importance of differentiating between the primary and secondary MS patients in future studies.

In summary, the preclinical evidence shows that ibudilast has a therapeutic potential in MS by inhibiting EAE in animal models and suppressing the secretion of pro-inflammatory cytokines. The clinical evidence demonstrates that ibudilast might be beneficial in the progressive forms of MS regarding brain atrophy, although more evidence is needed regarding its effectiveness in the rate of clinical progression of patients with progressive MS.

### 2.5. Other Neurodegenerative Diseases

There is also promising experimental evidence for PDE inhibitors in other neurodegenerative diseases, including HD. HD is an autosomal dominant, devastating neurodegenerative disorder, which is clinically characterized by chorea, cognitive decline, and psychiatric symptoms. PDE10A inhibition has been associated with increased cAMP and cGMP levels in the striatum of mouse models of HD, which is accompanied by decreased cortical and striatal cell loss and microglial activation [99]. In this regard, GSK356278, a PDE inhibitor, has been used in two phase I clinical trials [38]. However, the role of ibudilast in Huntington’s disease has not been investigated yet.

Wolfram syndrome is a rare childhood-onset autosomal recessive genetic disease; its main features are diabetes mellitus, as well as progressive optic nerve atrophy and hearing loss [100]. Wolfram syndrome is used as a model for neurodegeneration and diabetes. It is most often caused by mutations in the *Wolfram syndrome 1* (*WFS1*) gene [100]. Currently, there is no effective disease-modifying therapy, and the patients usually live until middle adulthood. A recent in vitro study demonstrated that the knock-out of *WFS1* in rat insulinoma (INS1) cells resulted in increased resting calcium levels in the cytosol, the downregulation of calcium signaling and reduced insulin secretion [101]. WFS1 or neuronal calcium sensor-1 (NCS1)—WFS1′s interacting partner—overexpression could reverse these observed deficits [101]. Interestingly, ibudilast and calpain inhibitor XI could also restore calcium homeostasis, cell viability and insulin secretion in this study [101]. Although the molecular mechanisms mediating these activities have not been investigated, it was proposed that ibudilast may normalize the calcium levels by interacting with NCS1 [101,102]. In addition, cAMP is majorly implicated in the calcium pathways, as well as in the insulin secretion and cell viability of the β cells [103]. This mechanism could be also involved in the neuroprotective effects of ibudilast in other neurodegenerative diseases, and its effects in mediating calcium signaling pathways in AD, MS and ALS remain to be explored.

Glaucoma, which is considered to be a neurodegenerative disease, is characterized by the selective loss of retinal ganglion cells, resulting in irreversible blindness [104]. Although the exact pathogenesis of the disease is not fully understood, neuroinflammation is critically implicated in its pathophysiology [104]. An in vivo study indicated that the intraocular administration of ibudilast in the rat models of ocular hypertension was associated with reduced microglia activation in the retina and optic nerve, resulting in reduced pro-inflammatory cytokines and gliosis, increased survival and restored axonal degeneration via the upregulation of cAMP/PKA signaling pathway [105]. This evidence suggests that ibudilast may also exert neuroprotective properties in glaucoma, and its therapeutic potential against this disease deserves further study.

Table 3 summarizes preclinical evidence on the role of ibudilast in neurodegenerative diseases.

## 3. Challenges and Future Perspectives

Despite the promising preclinical evidence about the role of ibudilast in a broad range of neurodegenerative disorders, the results of most of the clinical trials are not as they were expected to be (Table 4). The successful translation of the preclinical evidence to the clinical world is very challenging. The weak clinical evidence of the role of ibudilast in neurodegenerative diseases might be explained by several reasons. First, there is still no animal model that can effectively reflect the pathophysiology of each of the neurodegenerative diseases. Also, we still do not completely understand neuronal cell death in humans, which might differ from that in other animal species and patients with neurodegenerative diseases who comprise rather a biologically and clinically highly heterogeneous group [68]. Therefore, the careful selection of an appropriate subgroup of patients for each disorder and each drug candidate may represent a more promising approach towards the personalized treatment. Therapeutic strategies mainly acting in an anti-inflammatory manner may be more useful for the subgroups of patients with a “more prominent neuroinflammatory profile”, for example. The emerging development of biomarkers might help towards this direction in the future.

Regarding safety and tolerability, some of the abovementioned clinical trials were prematurely terminated because of limited tolerability and adverse events occurring. Finding the optimum dosing is a very important issue since inappropriately high doses may result in adverse events, while lower than optimal doses may result in inefficacy. Even though severe adverse events related to the drug have not been reported in most of the studies, gastrointestinal symptoms, headaches, and fatigue were very common, and these were related to low tolerability. An important limitation is the fact that PDEs are expressed in many human tissues [38]. Therefore, the development of novel selective PDE inhibitors with tissue- and even cell-specificity for specific treatment aims in the field of neurodegenerative diseases might overcome this important limitation. For example, based on the known levels of PDE expression in the brains of patients with AD, PDE8B, PDE4D1 and PDE4D2 represent appropriate targets [62]. Additionally, given the fact that PDE1 expression is relatively high in the hippocampus and frontal cortex, it may be another potential target candidate [62]. On the contrary, targeting PDE3 or PDE5, which display a relatively low expression in the brain tissue, may not be optimal to do [62]. However, a reduced expression or activity of a specific PDE family or subtype may represent a compensatory mechanism; in this case, further additional PDE inhibition may possibly deteriorate neurodegeneration. Therefore, specific PDE subtype targets should be carefully selected considering, also, the potential of adverse events occurring [62]. Another suggested strategy to mitigate the occurrence of adverse events is the combination of different PDE inhibitors, such as the use of low doses of PDE4 and PDE5 inhibitors [106], which may act in an additive or synergistic manner. This approach may allow for the use of lower doses of the separate PDE inhibitors, thereby preventing the occurrence of possible adverse events [62].

Furthermore, determining the optimal time for treatment is of paramount importance. It is generally well accepted that the earlier any potential disease-modifying treatment could be administered, the better the chance of slowing or preventing the progression of the neurodegenerative disease is [62]. As abovementioned, some in vivo studies described herein showed that ibudilast was ineffective when it was given after the onset of the clinical signs. For instance, it was shown that the preventive—but not the therapeutic—delivery of rolipram could ameliorate EAE in rats [91]. Currently, there are biomarkers that could help us to identify the cases of preclinical stages of AD-related dementia, including PET amyloid and CSF amyloid beta, phospho-tau, and total tau [1,4,9]. In addition, asymptomatic individuals with autosomal dominant gene mutations known to cause AD (*PSEN1, PSEN2, APP*) [4] represent another category that could benefit from the treatment at the preclinical stages. It can be speculated that clinical trials with ibudilast at the preclinical stages of neurodegenerative diseases might possibly show more promising results.

Ibudilast may also act in types of cells outside the CNS, such as circulating white blood cells [37], platelets and endothelial cells [107], as well as non-neuronal tissues. These activities might have implications for neurodegenerative diseases too. For instance, ibudilast can affect the levels of endothelial leukocyte adhesion molecules (P-selectin, vascular adhesion molecule 1 (VCAM-1) and intracellular adhesion molecule 1 (ICAM-1)) in the cerebral aneurysms of rats [28]. P-selectin has been shown to be reduced in the plasma of patients with AD [108], and higher VCAM-1 and ICAM-1 levels have been observed in AD compared to those in MCI [109]. Ibudilast can also downregulate matrix metalloproteinase-9 (MMP-9) [28]. It is well known that MMP-9 plays a significant role in neurodegeneration [110]. Hence, the P-selectin, VCAM-1, ICAM-1 and MMP-9-related mechanisms of ibudilast may apply for the neurodegenerative diseases too, although this hypothesis needs to be further investigated.

Furthermore, as mentioned above, apart from PDEs, ibudilast can also inhibit MIF and TLR-4. It is not fully elucidated which of the molecular and cellular effects of ibudilast are mediated through PDE inhibition in neurodegenerative diseases. Based on the existing evidence in Figure 1, we suggest which mechanisms might be mediated by PDE inhibition. Since the TLR-4 pathway implicates NF-κB, IRAK1 and TRAF6, it is possible that TLR-4 blocking may be at least partially responsible for the regulation of these factors by ibudilast. However, further evidence is needed in order to clarify the exact mechanisms via which ibudilast exerts its specific cellular and molecular effects in case of neurodegenerative diseases.

Caution is also needed for specific subpopulations. For instance, ibudilast was demonstrated to exhibit less tolerance among the patients with diabetes [47]. Furthermore, in the SPRINT-MS clinical trial, the eligibility criteria included an age between 21 and 65 years old like most of the older relevant studies in progressive MS [18,19]. Although the patients with relapsing remitting MS are most often of young ages, the patients with progressive MS may often be more than 65 years of age. Given the common comorbidities and the innate differences in the immune system during ageing [111], caution is needed for the future use of ibudilast in this subgroup. Active infections, such as chronic hepatitis, inflammatory or autoimmune conditions, as well as the concurrent use of immunomodulating drugs were the exclusion criteria in most of the clinical trials described above [18]. The patients with neurodegenerative diseases are often of an old age, and they often have several comorbidities. Therefore, the possible future clinical use of this drug in the real world for AD, PD or ALS requires additional considerations.

Notably, ALS patients carrying *C9ORF72* repeat expansion exhibit higher levels of microglial pathology in the motor cortex and the medulla compared to that of non-*C9ORF72* repeat expansion carriers [78]. Therefore, it could be hypothesized that drugs targeting the inflammatory pathways in ALS, such as ibudilast, may be more effective in selective genetic cases of ALS, including cases with *C9ORF72* repeat expansion. Since ALS is a highly heterogeneous disease, future clinical studies including those investigating ibudilast should target specific subpopulations of ALS patients that are more likely to respond to each proposed therapy.

## 4. Conclusions

In conclusion, the accumulating preclinical evidence shows that ibudilast may act neuroprotectively in neurodegenerative diseases by suppressing neuroinflammation, inhibiting apoptosis, regulating the mitochondrial function, and affecting the ubiquitin–proteasome and autophagosome–lysosome pathways as well as attenuating oxidative stress. The clinical trials in ALS and progressive MS also show some promising results, although further evidence is needed for evaluating its clinical effectiveness and safety. The development of selective PDE inhibitors, as well as the selection of the appropriate subgroup of participants in future relative clinical trials may aid in us in obtaining a better understanding of the therapeutic potential of ibudilast in neurodegenerative diseases.

## Figures and Tables

**Table 1:** Different PDE families, their tissue and organ distribution and their main inhibitors, focusing on those mentioned in the main text.

PDE Type	Distribution	Inhibitors	Reference
PDE1	Heart, lungs, brain, smooth muscle.	Ibudilast, nimodipine, dioclein, IC86340, IC224, IC295.	[22,40]
PDE2	Heart, kidneys, brain, platelets, adrenal glands, lungs, liver, endothelial cells.	Ibudilast, oxindole, EHNA, ND7001, BAY-60–7750, PDP, IC933.	[22,40,45]
PDE3	Heart, kidneys, brain, lungs, smooth muscle, liver, platelets, adipocytes, immune cells.	Ibudilast, cilostazol, milrinone, cilostamide, siguazodan.	[22,40,56,58,59]
PDE4	Heart, kidneys, brain, platelets, Sertolli cells, liver, smooth muscle, lungs, endothelial cells, immune cells.	Ibudilast, rolipram, cilomast, roflumilast, NCS 613.	[22,40,45,55,57]
PDE5	Platelets, heart, lungs, smooth muscle, brain, endothelial cells.	Ibudilast, DMPPO, zaprinast, vardenafil, sildenafil, tadalafil	[40,45]
PDE6	Lungs, pineal gland, photoreceptors.	DMPPO, zaprinast, sildenafil, vardenafil.	[40]
PDE7	Heart, skeletal muscle, T lymphocytes, kidneys, brain, pancreas.	IC242, BRL 50481, ASB16165.	[40]
PDE8	Brain, eyes, testes, liver, heart, skeletal muscle, kidneys, thyroid, ovaries, T lymphocytes.	PF-04957325.	[40,42]
PDE9	Lungs, kidneys, liver, brain.	PF-04447943, BAY-73–6691	[40]
PDE10	Brain, testes, thyroid.	Ibudilast, MP-10, Papaverine, TP-10.	[20,40,66,67]
PDE11	Heart, liver, skeletal muscle, pituitary gland, prostate.	Ibudilast, non-selective.	[20,40]

**Table 2:** Preclinical or clinical evidence on different PDE inhibitors—other than ibudilast—for neurodegenerative diseases, their PDE targets and main mechanisms of action.

PDE Inhibitor	PDE Target	Clinical Trials	Main Effects and Mechanism of Action in Neurodegenerative Diseases	Reference
**Alzheimer’s Disease**				
Rolipram	PDE4	-	Inhibition of Aβ-mediated cognitive decline, via the regulation of neuroinflammatory and apoptotic responses in rats through cAMP/CREB signaling.	[55]
Zatomilast	PDE4	Phase 1 clinical trials (NCT02648672, NCT02840279, NCT03030105); Phase 2 clinical trial (NCT03817684).	Improvement of memory, prevention of the loss of dendrites and spine density, inhibition of amyloid-beta-induced reduction of CREB, BDNF and NGF in the hippocampus of mice models of AD.	[57]
Cilostazol	PDE3	Randomized, placebo-controlled phase 4 clinical trial (NCT01409564).	Prevention of amyloid-beta-induced oxidative stress and memory impairment.	[58]
		-	Prevention of APOE-mediated amyloid-beta aggregation in mice.	[59]
		-	Induction of proteasome-mediated proteolysis, suppression of tauopathy and attenuation of cognitive impairment.	[60]
		-	Regulation of autophagy by upregulating SIRT1, and enhancement of amyloid-beta clearance and cell viability.	[61]
**Parkinson’s disease**				
Rolipram	PDE4	-	Inhibition of MPTP-induced dopamine loss in the striatum of mice, and prevention of dopaminergic neuronal loss in the SN.	[68]
FCPR16	PDE4	-	Prevention of the MPP+-induced reduction of oxidative stress and the potential of the mitochondrial membrane.	[69]
Zaprinast	PDE6, 5, 11 and 9	-	Prevention of cAMP and cGMP dysregulation in levodopa-induced dyskinesias in 6-OHDA-treated rat models of PD.	[70]
**Multiple Sclerosis**				
Rolipram	PDE4	Phase 2 clinical trial (NCT00011375).	Prevention of the clinical signs of demyelination in EAE rat models, reduction of TNF-α production in MBP-specific T cells.	[89]
		-	Reduction of TNF-α levels, prevention of clinical signs of MS and neuroimaging abnormalities on MRI in marmoset models of EAE.	[90]

**Table 3:** Preclinical evidence on the role of ibudilast in neurodegenerative diseases.

Neurodegenerative Disease	Type of Study	Model	Main Findings	Reference
Alzheimer’s disease	In vitro	Cultured hippocampal neurons from rats.	-Ibudilast could protect against glutamate-induced neurotoxicity and increase intracellular cAMP levels. -Ibudilast treatment was associated with reduced glutamate induced Ca^2+^ influx.	[23]
	In vivo	Sprague Dawley rats rat models	-Ibudilast could reverse the LPS- and INF-γ-induced inhibition of LTP in the CA1 region of hippocampus.	[44]
	In vivo	Amyloid-beta-injected mice mouse models of AD.	-Ibudilast pretreatment could prevent amyloid-beta-induced memory, spatial learning impairment, and neurotoxicity. -Ibudilast could inhibit the production of pro-inflammatory cytokines NF-κB p65 and TNF-α, prevent the activation of the pro-apoptotic protein caspase-3, and suppress the downregulation of the anti-apoptotic protein Bcl-2 in the cortex and hippocampus.	[34]
	In vivo	Fisher transgenic 344-AD rats.	-Long-term ibudilast treatment was associated with lower hippocampal-dependent spatial memory impairment, hippocampal amyloid-beta plaque deposition, tau paired-helical filament burden, and microgliosis. -RNA sequencing of hippocampal samples showed that ibudilast could affect the expression of the TLR, as well as the ubiquitin–proteasome pathways. -Ibudilast could downregulate the activity of IRAK1 by elevating the expression of IRAK3, affecting the levels of TRAF6 and possibly other TLR-related ubiquitin ligase.	[65]
Parkinson’s disease	In vivo	MPTP mouse models of PD.	-Pretreatment with ibudilast was associated with reduced astroglia activity and increased GDNF in the striatum. -Ibudilast could also suppress the production of pro-inflammatory cytokines, including IL-6, IL-1β and TNF-α. -Ibudilast did not alter the dopaminergic neuronal cell survival and TH levels in the striatum seven days after the acute MPTP insult in this study.	[66]
Amyotrophic Lateral Sclerosis	In vitro	HEK293 and NSC-34 cells.	-Ibudilast treatment could stimulate the clearance of SOD1 and TDP-43 aggregates, via induction of autophagy, increase in autolysosomes, and enhancement of lysosomal biogenesis, through the enhancement of the nuclear translocation of TFEB and the downregulation of the mTORC1. -Ibudilast could prevent TDP-43-induced neurotoxicity.	[81]
Multiple Sclerosis	In vivo	EAE rat models.	-Ibudilast pretreatment could prevent EAE in rats, although it could not alter the clinical course in case it was administered after the onset of the disease. -Ibudilast pretreatment could reduce neuroinflammatory responses in the spinal cord, inhibit MBP-induced T cell proliferation in the lymph nodes, reduce release of IFN-γ from T cells, and decrease secretion of TNF-α from macrophages.	[93]
Wolfram syndrome	In vitro	Rat insulinoma (INS1) cells.	-Knock out of WFS1 resulted in increased resting cytosolic calcium levels, downregulation of calcium signaling, and reduced insulin secretion. -Ibudilast and calpain inhibitor XI could also restore calcium homeostasis, cell viability and insulin secretion.	[101]
Glaucoma	In vivo	Rat models of ocular hypertension.	-Intraocular administration of ibudilast was associated with reduced microglia activation in the retina and optic nerve, resulting in reduced pro-inflammatory cytokines and gliosis, increased survival and restored axonal degeneration, via the upregulation of cAMP/PKA signaling pathway.	[105]

cAMP: cyclic adenosine monophosphate; LPS: lipopolysaccharide; INF-γ: interferon-gamma; LTP: long-term potentiation; IRAK1: interleukin 1 receptor associated kinase 1; IRAK3: interleukin-1 receptor-associated kinase 3; TNFR: tumor necrosis factor receptor; TNFR- associated factor 6: TRAF6; IL-6: interleukin 6; IL-1β interleukin-1-β; TNF-α: tumor necrosis factor; GDNF: Glial cell line-derived neurotrophic factor; TH: tyrosine hydroxylase; MPTP: 1-methyl-4-phenyl-1,2,3,6-tetrahydropyridine, TFEB: transcription factor EB; mTORC1: mammalian target of rapamycin complex 1; EAE: experimental autoimmune encephalitis; PKA: protein kinase A.

**Table 4:** Clinical trials investigating the role of ibudilast in neurodegenerative diseases.

Neurodegenerative Disease	Clinical Trial	Study Design	Study Objectives	Main Findings	Reference
**Amyotrophic Lateral Sclerosis**	NCT02238626	Randomized placebo-controlled Phase 1b/2a clinical trial	To evaluate the tolerability, safety, and clinical efficacy of ibudilast (60 mg/day) as an adjunct therapy to the standard riluzole treatment	-In the early cohort, ibudilast was safe and well-tolerated over a twelve-month period. -No significant difference in clinical progression was detected between ibudilast and placebo groups, as assessed by ALSFRS-R, hand-held dynamometry and ALSAQ-5. -Subgroup analysis demonstrated that ibudilast might provide more benefit for ALS patients with upper limb or bulbar onset, and possibly delay the progression of the disease if administered at an early stage, particularly if the onset of symptoms at screening was less than 17.1 months.	[35,82]
	NCT02714036	Open-label phase 1b clinical trial	To measure the impact of ibudilast on inflammation and axonal loss	-Ibudilast (up to 100 mg/day) was ineffective in inhibiting microglia activation in the primary motor cortex of ALS patients as evaluated by PBR28-PET over 12–24 weeks, and serum neurofilament light chain (NfL) levels, an indicator of neuronal axonal loss, remained unchanged over 36–40 weeks. -Most participants experienced at least one possibly ibudilast-related adverse event: about one-third of the patients required dosage reduction, while about another one-third discontinued ibudilast treatment because of ibudilast-related adverse events.	[49]
	NCT04057898	Phase 2b/3 randomized, double-blind, placebo-controlled clinical trial	To evaluate the safety, tolerability, and efficacy of ibudilast (up to 100 mg/day) for twelve months, followed by an open-label extension phase for six months in patients with ALS		Ongoing
**Progressive Multiple Sclerosis**	NCT01982942	Phase 2 randomized placebo-controlled clinical trial	To evaluate the safety, tolerability, and activity of ibudilast administered twice daily over a 96- week period in subjects with primary or secondary progressive multiple sclerosis	-Ibudilast (up to 100 mg/day) over a period of 96 weeks was associated with slower progression of the whole-brain atrophy and gray matter atrophy of patients with primary and secondary progressive MS. -Ibudilast was not associated with fewer new or enlarging T2-weighted or new T1-weighted MRI lesions. -Ibudilast treatment could also potentially attenuate retinal thinning on OCT. -Disability progression was similar between the ibudilast and placebo groups. -No significant alterations in NfL levels in the serum and CSF between ibudilast and placebo groups. -Most common adverse events in this study were gastrointestinal complains, headache, and depressive symptoms. -The overall treatment effect of ibudilast in brain atrophy was mainly driven by patients with primary progressive MS and not secondary progressive MS.	[18,36,95,96,97]

ALSFRS-R: ALS Functional Rating Scale-revised; ALSAQ-5: ALS Assessment Questionnaire-5 items; MS: multiple sclerosis; OCT: optical coherence tomography; PET: positron emission tomography; NfL: neurofilament light; MRI: magnetic resonance imaging; CSF: cerebrospinal fluid.

## Data Availability

Not applicable.
